# Supported treadmill training to establish walking in non-ambulatory patients early after stroke

**DOI:** 10.1186/1471-2377-7-29

**Published:** 2007-09-06

**Authors:** Louise Ada, Catherine M Dean, Meg E Morris

**Affiliations:** 1 Physiotherapy, Faculty of Health Sciences, The University of Sydney, PO Box 170, Lidcombe, NSW, 1825, Australia; 2 School of Physiotherapy, The University of Melbourne, 200 Berkeley St, Melbourne, Victoria, 3010, Australia

## Abstract

**Background:**

It has been reported that only half of the non-ambulatory stroke patients admitted to inpatient rehabilitation in Australia learn to walk again [1]. Treadmill walking with partial weight support via an overhead harness is a relatively new intervention that is designed to train walking. The main objective of this randomised controlled trail is to determine whether treadmill walking with partial weight support via an overhead harness is effective at establishing independent walking (i) more often, (ii) earlier and (iii) with a better quality of walking, than current physiotherapy intervention for non-ambulatory stroke patients.

**Methods:**

A prospective, randomised controlled trial of inpatient intervention with a 6 month follow-up with blinded assessment will be conducted. 130 stroke patients who are unable to walk independently early after stroke will be recruited and randomly allocated to a control group or an experimental group. The control group will undertake 30 min of routine assisted overground walking while the experimental group will undertake 30 min of treadmill walking with partial weight support via an overhead harness per day. The proportion of participants achieving independent walking, the quality of walking, and community participation will be measured. The study has obtained ethical approval from the Human Research Ethics Committees of each of the sites involved in the study.

**Discussion:**

Given that the Australian population is ageing and people after stroke can expect to live for longer, attainment of safe, independent walking is more likely to be associated with long-term health and well being. In its National Research Priorities, the Government has recognised that it will be important to promote healthy ageing and that this endeavour will be underpinned by research. The results of this study will clearly identify effective intervention to establish early quality walking, thereby promoting an increase in community participation in the longer term.

**Trial Registration:**

The protocol for this study is registered with US NIH Clinical trials registry (NCT00167531)

## Background

It has been reported that only half of the non-ambulatory stroke patients admitted to inpatient rehabilitation in Australia learn to walk again [1]. Treadmill walking with partial weight support via an overhead harness is a relatively new intervention that is designed to train walking. In order to optimise the outcome of walking, practice is critical because skill in performance improves as a function of practice [2]. For non-ambulatory stroke patients, treadmill walking with partial weight support provides the opportunity to complete more walking practice than would be possible using assisted overground walking. There is evidence from systematic reviews that outcome following stroke is associated with the amount of practice undertaken [3,4]. However, Australian research has shown that little practice is completed in rehabilitation [5,6]. One of the barriers to completion of more walking practice in non-ambulatory stroke patients is marked muscle weakness and poor coordination, which results in an inability to practice the whole task. Even with the assistance of a therapist, they may not be able to complete even a few steps of overground walking. Treadmill walking with partial weight support via an overhead harness provides the opportunity to complete larger amounts of walking practice, eg, even if patients only walk for 5 min at a slow speed of 0.2 m/s supported on a treadmill, they will 'walk' 60 m.

The provision of weight support seems crucial since Visintin et al [7], in a randomised controlled trial, found a small beneficial effect when treadmill training was combined with partial weight support compared with no weight support. Moreover, treadmill walking with partial weight support via an overhead harness means that the therapist can provide the opportunity for subjects to complete large amounts of walking practice without contravening occupational health and safety standards in that the therapist is less likely to injure their back and the subject is less likely to fall. Furthermore, in using this equipment [8], we have developed innovative equipment and procedures to enable one therapist to deliver the intervention safely. For example, we have modified a chair to support the therapist while lifting the affected foot through, modified footwear to allow for easy lifting of the affected foot, and designed an attachment to support the affected hand.

The efficacy of treadmill walking with partial body weight support in non-ambulatory patients after stroke is unclear. A Cochrane Collaboration Systematic Review [9] concludes that there is as yet no definitive answer about whether this intervention helps more non-ambulatory patients learn to walk compared to assisted overground walking. There are randomised controlled trials examining whether treadmill walking and partial body weight support is effective in a mixed group of ambulatory and non-ambulatory patients [7,10-13]. The only RCTs which examine non-ambulatory patients, compare treadmill training and partial body weight support with alternative interventions such as a mechanised gait trainer [14] or aggressive bracing [15] rather than current practice. This situation has led to the Cochrane Collaboration Systematic Review [9] to recommend that separate, large number, high quality studies for non-ambulatory patients be undertaken to examine the efficacy of treadmill walking with partial body weight support after stroke.

The main objective of this randomised controlled trail is to determine whether treadmill walking with partial weight support via an overhead harness is effective at establishing independent walking (i) more often, (ii) earlier and (iii) with a better quality of walking, than current physiotherapy intervention for non-ambulatory stroke patients. The study targets stroke patients who are unable to walk independently on admission to rehabilitation. It will determine the efficacy of treadmill walking with partial weight support via an overhead harness on the establishment and quality of independent walking. Furthermore, by assessing community participation six months later, it will evaluate the longer-term effect of this intervention.

## Methods

### Design

A prospective, randomised controlled trial will be carried out. 130 subjects will be randomly allocated into either an experimental group (treadmill walking and partial weight support with one therapist) or a control group (assisted overground walking with one therapist) by a recruiter blinded to the sequence of group allocation. All outcome measures and data analysis will be completed by a researcher who is blinded to participant group allocation. The study has obtained ethical approval from the Human Research Ethics Committees of each of the sites involved in the study.

### Participants

Stroke patients will be screened and invited to participate if they:

• are within 3 weeks of their first stroke

• are aged between 50 and 85 years of age

• are diagnosed clinically with hemiparesis or hemiplegia of acute onset, and

• are non-ambulatory defined as scoring 0 or 1 on the Motor Assessment Scale for stroke.

Participants will be excluded if they:

• have clinically evident brainstem signs

• have severe cognitive and/or language deficits which preclude them from following instructions in training sessions

• have unstable cardiac status which would preclude participation in a rehabilitation program, or

• have any pre-morbid history of orthopaedic conditions of the lower limbs which would preclude them from relearning to walk.

The presence of sensory loss, neglect and/or spasticity will not be exclusion criteria. However, their severity will be recorded using the Nottingham Sensory Assessment for sensory loss, the line bisection test for neglect, and the Ashworth Scale for spasticity. In addition, information about site and size of lesion will be collected.

### Randomization

Participants will be randomised into an experimental or a control group. We will stratify the randomisation. First, given the potential confounder of site, participants at each site will be randomised separately. Second, at each site, participants will be stratified according to initial level of motor disability since it has been found to affect outcome [16]. Since all the participants will be unable to walk on admission to the study, sitting balance will be used to stratify the allocation of participants to groups because it has been found to be a useful prognostic indicator of walking outcome [17-19]. Therefore, participants will be stratified according to Item 3 (Sitting Balance) of the Motor Assessment Scale for stroke [20] so that those with a score of 0–3 will be classified as severely disabled and those with a score of 4–6 will be classified as moderately disabled. Within each of the two strata (moderate versus severe level of disability), participants will be allocated randomly to one of two groups, the experimental group or the control group. Random permuted blocks will be used so that after every block (of 6–10 participants), the experimental and control groups will contain equal numbers. In summary, stratification will occur according to site (three sites) and level of disability (two levels). Therefore, there will be 6 strata and participants will be randomised separately within each stratum. The random sequence of group allocation will be concealed from the person recruiting participants.

### Intervention

Both the experimental and the control group will undergo a maximum of 30 minutes per day of walking practice with assistance from one therapist, five days a week until they walk or until discharge from rehabilitation. The total daily time of intervention will be 30 minutes from beginning (ie, from when the participant is in the gym) to end (ie, when the participant is back in the wheelchair). This time therefore includes transferring, putting on aids or setting up equipment, ie, training does not have to be continuous so that rests may be taken. The amount of assistance during walking will be standardised to one therapist, however, additional help will be allowed during setting up walking (ie, getting the participant onto the treadmill for treadmill walking or into standing for overground walking). The rationale for this protocol is based on clinical observation of how much time and how many therapists are currently used in trying to get a non-ambulatory person to walk. Other intervention involving lower limb function, (ie, strengthening exercises, practice loading the affected leg during activities such as sitting, standing up and standing) will be standardised to 60 min per day. No other part of the multidisciplinary rehabilitation program will be controlled. Randomization should ensure that any effect of other interventions will be the same for both groups, therefore, other therapies will not be withheld.

#### Experimental group

Training for the *experimental *group will primarily involve walking on a treadmill supported in a body harness. Treadmill training with partial weight support via an overhead harness will be conducted using commercially available systems such as the Spacetrainer (TR Equipment, Tranas, Sweden) and the Lite-Gait (Mobility Research, USA). These systems have an access ramp so that a wheelchair can be wheeled onto the treadmill and an automatic lifter so that the harness can be prefitted in sitting or lying and the patient lifted into standing. In addition, there is good access to the patient's legs and the treadmills can run extremely slowly allowing adequate time to assist the legs to swing through.

There will be guidelines to determine the progression of training both in terms of increasing treadmill speed and reducing weight support. At the start, support from the harness will be as little as possible but up to a maximum of 40% of body weight since Hesse et al [21,22] have found this to be the maximum support which does not dramatically alter the kinematic and kinetic features of walking. The actual weight relief will be determined by observation of whether the knee can extend in midstance. If the knee remains flexed, then the affected lower limb muscles are too weak to support the body weight and indicates that more weight relief is required. At the start, the speed of the treadmill will be as fast as comfortable while still maintaining a reasonable step length. If a participant is too disabled to walk on a treadmill moving at 0.1 m/s with the assistance of one therapist, they will walk on the spot practising lifting their feet rhythmically. When participants attain a speed of 0.4 m/s, a reduction in weight support will occur if participants can (i) swing their affected leg through without help, (ii) maintain a straight knee during stance phase without hyperextension, and (iii) maintain an adequate step length (rather than a high cadence) without help. These guidelines have been tested for feasibility and published [8]. Information describing the specific features of the training session (such as treadmill speed, amount of weight support, distance walked, assistance required) will be recorded to monitor adherence to the guidelines and to be able to describe the intervention accurately.

#### Control group

Training for the *control *group will involve current practice of assisted overground walking. If a participant is too disabled to walk with the help of one therapist, they will practise stepping forwards and backwards or standing with a knee splint and practising shifting weight from leg to leg. Aids such as knee splints, ankle-foot orthoses, parallel bars, forearm support frames and walking sticks can be utilised as part of training. Training aims to produce independent walking. Therefore, progression of training encompasses both increasing speed and reducing assistance from both aids and the therapist. Information describing the specific features of the training session (such as use of aids, distance walked, assistance required) will be recorded to monitor adherence to the guidelines and to be able to describe the intervention accurately.

The end-point of the training phase of the study will be either the attainment of independent walking or discharge from the rehabilitation unit. The end-point of the follow-up phase of the study will be 6 months after admission to the study.

### Measurement

The initial outcome measures will be:

#### Proportion of participants achieving independent walking

Independent walking will be operationally defined as 'being able to walk 15 m continuously barefoot across flat ground without any aids'. Participants will be tested once a week in the morning (ie, before the training session). Participants will continue to be tested until they achieve independent walking or are discharged.

#### Quality of independent walking

Quality of walking will be measured by quantifying parameters such as speed, affected and intact step length, step width, and cadence. These parameters are the result of the timing and magnitude of the angular displacements during walking and are therefore global measures which reflect qualitative aspects of walking. When participants achieve independent walking, their overground walking will be measured by placing markers on the heels of both the unaffected and affected legs, so that step length of both the affected and unaffected leg, walking speed, cadence, and step width can be determined.

The 6-month outcome measures will be:

#### Proportion of participants achieving independent walking

#### Quality of independent walking

#### Community participation

Two aspects of community participation will be measured. First, mobility status will be assessed using the 6-min walk test, number of falls since discharge, a self-efficacy questionnaire about walking capability, and the Adelaide Activities Index. Second, living arrangements will be assessed by recording the type of residence and the amount of support within the residence.

### Sample size

130 participants will be recruited. The sample size has been calculated to reliably detect a treatment effect size of a 25% increase in proportion of independent walkers with 80% power at a two-tailed significance level of 0.05. For non-ambulatory patients, it takes an average of 3 months to achieve independent walking with about 50% walking independently at six weeks [1]. We are interested in being able to detect a 25% increase, from 50% to 75%, proportion of non-ambulatory patients walking independently by six weeks. Only an effect such as this is clinically significant enough to warrant a change in the implementation of services which would involve the re-education of physiotherapists and the expense of purchasing a treadmill and overhead harness system. The smallest number of participants to detect this difference between two proportions estimated from independent samples is 65 participants per group, ie, 130 participants in total [23]. However, since the analysis of the data is survival curve analysis, there is greater power than the calculation would suggest because the effect of dropouts is minimal in this analysis. For example, in a pre-post design trial of 6 weeks intervention, if a subject dies or is lost to follow-up (ie, drops out) at 5 weeks, there is no measurement at the post-test on which to perform an analysis. In this trial, which ascertains once a week whether independent walking has been established, they would be censored at five weeks so that they no longer form part of the total sample. However, this participant's data would be available for the five weeks they participated in the trial thereby minimising the effect of their dropping out.

In addition to, but separate from, the difference in the proportion of participants walking between the groups, there may also be a difference in the quality of walking. On the assumption that 20% of participants may be lost to follow-up and that 80% of participants entering rehabilitation achieve basic independent walking [1], there are likely to be 84 participants with "quality of walking" data at 6 months. Goldie et al [24] has suggested that a minimum difference in walking speed worth detecting is 0.2 m/s. The walking speed of a population of stroke patients who have recently completed rehabilitation [25] on entry to a randomised controlled trial was 0.56 (SD, 0.27) m/s using measurement procedures similar to the present proposal. 60 participants are needed to detect a treatment effect size of 0.2 m/s difference in walking speed between the groups at 6 months with 80% power at a two-tailed significance level of 0.05 therefore 84 participants gives over 90% power.

### Statistical analysis

The proportion of independent walkers and the time to achieve independent walking will be compared between the two groups using the logrank test, in which those who do not achieve independent walking are censored at the time they are discharged. Survival analysis using Cox's regression will be used to compare the times in the two groups while allowing for possible confounding variables, such as other interventions received, and baseline sitting balance.

The five variables that reflect the quality of walking: speed, affected and intact step length, step width and cadence, will be compared between the two groups using Student's t-test, or Wilcoxon's rank-sum test for variables that are clearly not Normally distributed.

The four variables that reflect the mobility status: 6-min distance, number of falls since discharge, self-efficacy questionnaire about walking capability, and the Adelaide Activities Index will be compared between the two groups using Student's t-test, or Wilcoxon's rank-sum test for variables that are clearly not Normally distributed.

The two variables which reflect living arrangements: type of residence and the amount of support within the residence, will be analysed descriptively.

Descriptive data about lesion, neglect, spasticity and sensation will be used in post-hoc multiple regression analysis to examine if these factors affected walking outcome.

## Discussion

Australia, like many other developed nations, is undergoing a major demographic shift involving significant growth in the aged population. The Australian Government has recognised that a revolution is underway at the end of the life cycle. In its National Research Priorities, the Government has recognised that it will be important to promote healthy ageing and that this endeavour will be underpinned by research. Only by establishing evidence-based interventions, such as the one outlined in this project, will the severity of many health problems be reduced. In economic terms, if non-ambulatory patients do not learn to walk after their stroke, it is likely that they will require assisted care, placing a high burden on the community. Furthermore, given that independence in walking is a major factor in the decision to discharge patients from inpatient care, earlier independent walking should result in a reduction in length of hospital stay which should result in cost saving. The Queensland Health Report "Hospital benchmarking pricing model" has estimated the cost of a day in hospital for a neurological patient at $880. In summary, this study has the potential to reduce disability following stroke by improving the outcome of walking and thereby to reduce the burden of this condition on the community. Increasing the number of people who can walk after stroke should reduce the demand for nursing home placement.

## Competing interests

The author(s) declare that they have no competing interests.

## Authors' contributions

LA and CD conceived this study. All authors contributed to the design and procurement of funding. LA and CD drafted the manuscript and all authors have contributed to the manuscript and have read the manuscript.

## Pre-publication history

The pre-publication history for this paper can be accessed here:


